# Size for gestational age affects the risk for type 1 diabetes in children and adolescents: a Swedish national case–control study

**DOI:** 10.1007/s00125-021-05381-y

**Published:** 2021-02-05

**Authors:** Nina Lindell, Marie Bladh, Annelie Carlsson, Ann Josefsson, Karin Aakesson, Ulf Samuelsson

**Affiliations:** 1 Department of Obstetrics and Gynecology, Linköping University, Linköping, Sweden; 2 Department of Biomedical and Clinical Sciences, Linköping University, Linköping, Sweden; 3 Department of Clinical Sciences, Skåne University Hospital, Lund University, Lund, Sweden; 4 Department of Pediatrics, Ryhov County Hospital, Jönköping, Sweden; 5 Department of Pediatrics, Linköping University, Linköping, Sweden

**Keywords:** Age at onset, Children, Epidemiology, Large for gestational age, Risk factor, Small for gestational age, Type 1 diabetes

## Abstract

**Aim/hypothesis:**

Environmental factors are believed to contribute to the risk of developing type 1 diabetes. The aim of this study was to investigate how size for gestational age affects the risk of developing childhood type 1 diabetes.

**Methods:**

Using the Swedish paediatric diabetes quality register and the Swedish medical birth register, children with type 1 diabetes diagnosed between 2000 and 2012 (*n* = 9376) were matched with four control children (*n* = 37,504). Small for gestational age (SGA) and large for gestational age (LGA) were defined according to Swedish national standards. Data were initially analysed using Pearson’s *χ*^2^ and thereafter by single and multiple logistic regression models.

**Results:**

An equal proportion of children were born appropriate for gestational age, but children with type 1 diabetes were more often born LGA and less often born SGA than control children (4.7% vs 3.5% and 2.0% vs 2.6%, respectively, *p* < 0.001). In the multiple logistic regression analysis, being born LGA increased (adjusted OR 1.16 [95% CI 1.02, 1.32]) and SGA decreased (adjusted OR 0.76 [95% CI 0.63, 0.92]) the risk for type 1 diabetes, regardless of maternal BMI and diabetes.

**Conclusions/interpretation:**

Size for gestational age of Swedish children affects the risk of type 1 diabetes, with increased risk if the child is born LGA and decreased risk if the child is born SGA. Being born LGA is an independent risk factor for type 1 diabetes irrespective of maternal BMI and diabetes. Thus, reducing the risk for a child being born LGA might to some extent reduce the risk for type 1 diabetes.

**Graphical abstract:**

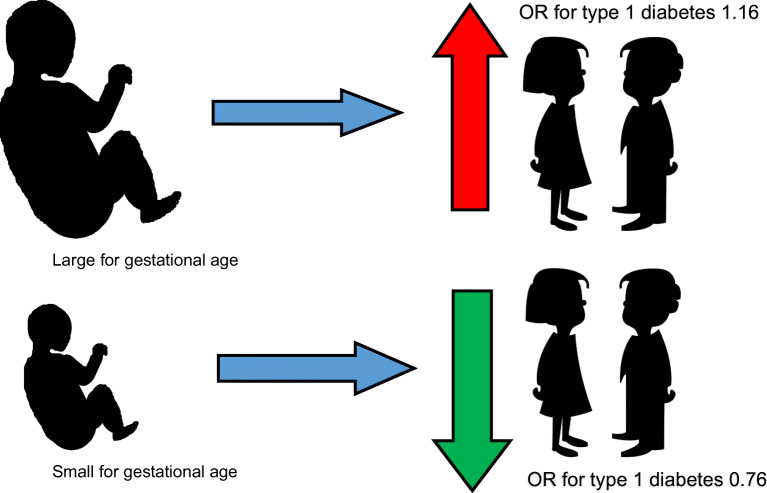

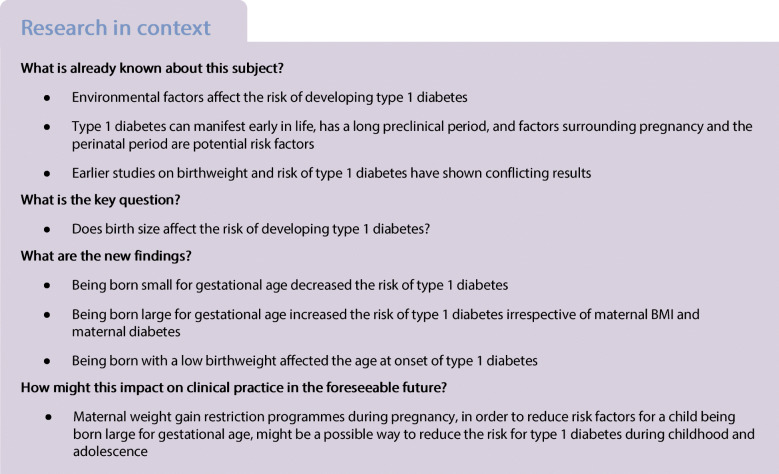



## Introduction

The incidence of type 1 diabetes, one of the most common chronic diseases among children and adolescents, has rapidly increased over the last decades. Sweden has one of the highest incidences in the world, surpassed only by Finland [1]. Although there is a known genetic association between type 1 diabetes and certain HLA-alleles, such as DQ2 and DQ8 [2], the cause of the disease is mostly unknown. It is generally believed that both genetic and environmental factors contribute to the pathogenesis and that environmental factors are responsible for the steep increase in incidence [3]. Since type 1 diabetes can manifest early in life, and there is a long preclinical phase before clinically overt disease develops [4], perinatal factors concerning the child have been proposed to contribute to the pathogenesis [5].

There is a great variation in the prevalence of children born small for gestational age (SGA) and large for gestational age (LGA) worldwide and it has changed over time. In most countries there has been an increase in the incidence of children born LGA in recent decades, though in the last few years there have been reports of a shifting trend. On the other hand, the incidence of children born SGA has been stable or decreasing over recent decades [6].

The results of previous studies have been conflicting. A cohort study from the UK, including 991 children with type 1 diabetes, showed an increased risk for type 1 diabetes if the child had a birthweight >4000 g vs <3000 g (RR 1.47 [95% CI 1.16, 1.85]) [7], and a meta-analysis of 11 studies (in total 7491 cases) showed an increased risk for type 1 diabetes if the child was born >4000 g vs <4000 g (OR 1.17 [95% CI 1.09, 1.26]) [8]. A Swedish case–control study, including 4584 cases, found that the OR for type 1 diabetes was 1.20 (95% CI 1.02, 1.42) if the child was born LGA [9]. A high birthweight has also been shown to be associated with a higher weight later in life [10]. Other studies have shown that childhood obesity and a rapid linear growth in childhood increases the risk of type 1 diabetes [11]. A low birthweight, or to be born SGA, has been shown to correlate negatively with the risk of type 1 diabetes [9, 12, 13]. However, many smaller case–control studies have failed to show any significant correlation between birthweight/LGA/SGA and subsequent risk of type 1 diabetes [14–17]. In addition, a low birthweight (<2500 g) has been shown to correlate with earlier onset of type 1 diabetes [18]. Several studies on this subject have used absolute birthweight, but it is important to correct for gestational age when looking at birthweight since there is a strong correlation between the two [19].

When studying size for gestational age, it is important to adjust for maternal factors known to affect birthweight such as maternal age, BMI, parity, gestational weight gain, maternal smoking and maternal diabetes [6, 20].

We hypothesise that being born LGA or SGA affects the risk of the child later being diagnosed with type 1 diabetes. Our large and nationwide body of material gives us an exceptional opportunity to investigate the potential relationship between these factors in a case–control study. Hence, the aim of this case–control study is to investigate whether size for gestational age affects the future risk of type 1 diabetes, and the age at onset of type 1 diabetes, and to determine whether there is a sex difference or not.

## Methods

### Registries

#### SWEDIABKIDS

The Swedish paediatric diabetes quality register (SWEDIABKIDS) (http://www.ndr.nu/#/om-swediabkids) was introduced stepwise in Sweden during 2000–2007, and since 2007 all 42 paediatric clinics in Sweden have reported to the register data on approximately 99% of children and adolescents (0–18 years of age) with diabetes in Sweden. All newly diagnosed children and adolescents with type 1 diabetes and their parents give oral informed consent before being registered, and from the time of diagnosis all data are prospectively reported. On 31 December 2019, the register included data from 472,369 outpatient visits, involving a total of 20,966 patients (data from K. Aakesson, one of the keepers of the register). SWEDIABKIDS is financially supported by the Association of Local Authorities and Regions (SALAR), which represents the professional, governmental and employer-related interests of Sweden’s municipalities, county councils and regions (http://skr.se/english).

#### MBR

The Swedish Medical Birth Register (MBR) is based on the medical charts from antenatal, obstetric and neonatal care and contains information on more than 98% of all births in Sweden since 1973. Where data are missing, this is due to hospital errors in reporting [21]. The mother’s personal identification number was used to collect information about her height, parity, weight in early pregnancy and at delivery, gestational week at childbirth and smoking habits. Also, information about the child’s weight, height and Apgar score at birth was collected as well as information about maternal diabetes corresponding to the diagnostic code O24 in ICD-10 (www.who.int/classifications/icd/en). In 1997, the MBR started to report the different types of diabetes, i.e. O24.0 pre-existing type 1 diabetes, O24.1 pre-existing type 2 diabetes and O24.4 gestational diabetes. Before that the diagnosis of diabetes was not always further specified. In the MBR, data are missing on maternal BMI in early pregnancy (*n* = 11,927), weight gain during pregnancy (*n* = 30,288), smoking during pregnancy (*n* = 2263), duration of pregnancy (*n* = 657), parity (*n* = 3), length at birth (*n* = 654) and birthweight (*n* = 132).

### Study population

The study population consists of children and adolescents (age 0–18 years) diagnosed with type 1 diabetes between January 2000 and October 2012 and registered in SWEDIABKIDS (*n* = 9376). Information from the MBR was added to the SWEDIABKIDS register using the child’s unique personal identification number. The children were born between 1982 and 2011; the majority between 1990 and 2011 (91%), and their age range at diagnosis was 0–18 years. As expected, there were more boys than girls (55.2% vs 44.8%). All children with diabetes were matched with four control children from the MBR with the same year and day of birth, same sex, and born in the same region of Sweden (*n* = 37,504). We excluded all children who were not singletons (*n* = 552; 454 control children and 98 children with type 1 diabetes).

The study was approved by the regional ethics review board in Linköping (Dnr 2011/381–31), prior to any data collection.

### Exposure variables

SGA was defined as a sex-specific birthweight <−2 SD of the mean weight for the gestational length. LGA was similarly defined as a birthweight >+2 SD of the mean weight for the gestational length according to the Swedish standard [22]. Children who were neither SGA nor LGA were considered appropriate for gestational age (AGA). Children with missing values on either birthweight or gestational age (*n* = 1237, 0.3%) were assumed to be AGA. Birthweight <1500 g was considered very low birthweight, 1500–2499 g low birthweight, 2500–3999 g normal birthweight and ≥4000 g macrosomia.

Children were categorised into four groups depending on gestational age at delivery; very preterm (<32 weeks of gestation), moderately preterm (32^0^ through 36^6^ weeks), term (37^0^ through 41^6^ weeks) and post term (≥42 weeks).

Women were categorised into four BMI groups according to the WHO classification of BMI cut-off values [23] and gestational weight gain was defined as inadequate, adequate or excessive according to the 2009 guidelines of the US Institute of Medicine and the US National Research Council [24]. Maternal smoking during pregnancy is a composite of women who smoked in early or late pregnancy or both in early and late pregnancy.

To further define the relationship between size for gestational age, maternal BMI and maternal diabetes, a variable with four categories was created (maternal BMI <25 kg/m^2^ and no maternal diabetes, maternal BMI ≥25 kg/m^2^ and no maternal diabetes, maternal BMI <25 kg/m^2^ and maternal diabetes of any kind, maternal BMI ≥25 kg/m^2^ and maternal diabetes of any kind).

### Statistical analyses

The *χ*^2^ test was used to analyse the differences in distributions between size for gestational age, birthweight and gestational age, as well as several maternal characteristics such as BMI, and the proportion of children who developed type 1 diabetes (Table 1). The *χ*^2^ test was also utilised to assess the association between age at onset of type 1 diabetes and gestational age and birthweight. Multivariate analysis encompassed multiple logistic regression in which the dependent variable was type 1 diabetes in the child and independent variables were size for gestational age, gestational age, maternal diabetes, maternal BMI in early pregnancy, maternal age at delivery and maternal smoking (Table 3). Missing data were only excluded in the regression analysis. All statistical analyses were performed using IBM SPSS Statistics, version 25 (IBM, Armonk, NY, USA), and a *p* value of <0.05 (two-sided) was considered statistically significant.

**Table 1:** Characteristics of children with and without type 1 diabetes and their mothers

Characteristic	All cases		
	Control children	Index children	*p* value^a^
	(*n* = 37,050)	(*n* = 9278)	
Size for age^b^			<0.001
AGA	34,789 (93.9)	8661 (93.3)	
SGA	962 (2.6)	183 (2.0)	
LGA	1299 (3.5)	434 (4.7)	
Gestational age			0.014
Very preterm (<32 weeks)	261 (0.7)	45 (0.5)	
Moderately preterm (32–36 weeks)	1799 (4.9)	501 (5.4)	
Term (37–42 weeks)	34,730 (93.9)	8680 (93.6)	
Post term (>42 weeks)	207 (0.6)	45 (0.5)	
Maternal diabetes			<0.001
No	36,733 (99.1)	9005 (97.1)	
Yes	317 (0.9)	273 (2.9)	
Maternal BMI^b^			<0.001
Underweight (<18.5 kg/m^2^)	936 (3.4)	198 (2.8)	
Normal weight (18.5–24.9 kg/m^2^)	18,217 (66.4)	4460 (64.1)	
Overweight (25.0–29.9 kg/m^2^)	6039 (22.0)	1611 (23.1)	
Obese (≥30.0 kg/m^2^)	2248 (8.2)	691 (9.9)	
Maternal diabetes and BMI			<0.001
No maternal diabetes and BMI <25	19,036 (69.4)	4551 (65.4)	
No maternal diabetes and BMI ≥25	8158 (29.7)	2191 (31.5)	
Maternal diabetes and BMI <25	118 (0.4)	107 (1.5)	
Maternal diabetes and BMI ≥25	129 (0.5)	111 (1.6)	
Maternal weight gain during pregnancy			0.063
Adequate	5061 (39.4)	1292 (40.4)	
Excessive	4664 (36.3)	1194 (37.3)	
Inadequate	3116 (24.3)	713 (22.3)	
Maternal smoking during pregnancy			<0.001
No	28,037 (79.7)	7300 (82.3)	
Yes	7158 (20.3)	1570 (17.7)	
Maternal age			0.048
13–29 years	20,445 (55.2)	5014 (54.0)	
>29 years	16,605 (44.8)	4264 (46.0)	

Data are given as *n* (%)

In the MBR, data are missing on maternal BMI in early pregnancy (*n* = 11,927), weight gain during pregnancy (*n* = 30,288), smoking during pregnancy (*n* = 2263), duration of pregnancy (*n* = 657), and birthweight (*n* = 132, used to calculate size for age)

^a^*χ*^2^ test: control children vs index children

^b^Statistically significant linear-by-linear association with the presence of type 1 diabetes in the children, all *p* < 0.001

**Table 2:** Characteristics of children born at term with and without type 1 diabetes

Characteristic	Children born at term		
	Control children	Index children	*p* value^a^
	(*n* = 34,981)	(*n* = 8741)	
Size for age			<0.001
AGA	33,035 (94.4)	8206 (93.9)	
SGA	742 (2.1)	153 (1.8)	
LGA	1204 (3.4)	382 (4.4)	
Birthweight			0.001
Very low (<1500 g)	7 (0.0)	2 (0.0)	
Low (1500–2499 g)	424 (1.2)	77 (0.9)	
Normal (2500–3999 g)	27,371 (78.4)	6724 (77.1)	
Macrosomia (≥4000 g)	7102 (20.3)	1919 (22.0)	

Data are given as *n* (%)

In the MBR, data are missing on birthweight (*n* = 132), a parameter used in calculating size for age

^a^*χ*^2^ test: control children vs index children

## Results

Most children were born AGA (93.8%), whereas 3.7% were born LGA and 2.5% were born SGA, with no significant sex differences. Children with type 1 diabetes were significantly more often born LGA and significantly less often born SGA than control children (4.7% vs 3.5% and 2.0% vs 2.6%, respectively, *p* < 0.001) (Table 1). Limiting the study population to only children born at term, their size for gestational age was a little more evenly distributed, but the statistical significance remained (Table 2). The same was true when using the broader definition of LGA (weight above the 90th percentile for that gestational age) and SGA (weight below the 10th percentile for that gestational age).

The absolute birthweight revealed that more children who later developed type 1 diabetes had a birthweight of ≥4000 g (macrosomia), and fewer were born with a low (1500–2499 g) or very low (<1500 g) birthweight (*p* < 0.001). After restricting the analysis only to children born at term, the difference remained (*p* < 0.001, Table 2). The proportion of children, regardless of sex, with a birthweight <2500 g increased with age at diagnosis; in the youngest age group (0–4 years) it was 2.0%, and this increased stepwise (5–9 years; 2.6% and 10–14 years; 2.8%) to 4.0% in the oldest age group (15–19 years). For children born SGA, the results did not reach statistical significance (*p* = 0.073).

Furthermore, children with type 1 diabetes more often had an older mother (age >29 years, 46.0% vs 44.8%, *p* = 0.048), whereas the control children more often had a mother who had smoked during pregnancy (20.3% vs 17.7%, *p* < 0.001). The gestational age also showed a slightly different distribution between children with type 1 diabetes and control children (*p* = 0.014), with more children who later developed type 1 diabetes having been born moderately preterm (5.4% vs 4.9%, Table 1).

A linear-by-linear association was found between type 1 diabetes and maternal BMI and the child’s size for gestational age and the child’s absolute birthweight.

### Regression analysis

In the multivariate analysis (Table 3), a child born LGA had an increased risk of developing type 1 diabetes (crude OR 1.34 [95% CI 1.20, 1.50]; adjusted OR 1.16 [95% CI 1.02, 1.32]), while a child born SGA was less likely to develop type 1 diabetes (crude OR 0.76 [95% CI 0.65, 0.90]; adjusted OR 0.76 [95% CI 0.63, 0.92]). To verify the presented findings, a sensitivity analysis excluding cases assumed to be AGA due to missing data was performed. This sensitivity analysis did not alter the findings, thus the results from the main analysis can be viewed as reliable.

**Table 3:** Logistic regression model with ORs for developing type 1 diabetes

Characteristic	All cases		
	Crude OR (95% CI)	Adjusted OR (95% CI)^a^	Adjusted OR (95%CI)^b^
Size for age			
AGA	Reference	Reference	Reference
SGA	0.76 (0.65, 0.90)	0.80 (0.66, 0.98)	0.76 (0.63, 0.92)
LGA	1.34 (1.20, 1.50)	1.15 (1.01, 1.31)	1.16 (1.02, 1.32)
Gestational age			
Very preterm (<32 weeks)	0.69 (0.50, 0.95)	0.70 (0.46, 1.07)	0.69 (0.47, 1.02)
Moderately preterm (32–36 weeks)	1.11 (1.01, 1.23)	1.10 (0.97, 1.24)	1.06 (0.94, 1.19)
Term (37–42 weeks)	Reference	Reference	Reference
Post term (>42 weeks)	0.87 (0.63, 1.20)	0.91 (0.62, 1.34)	0.92 (0.62, 1.35)
Maternal diabetes			
No	Reference	Reference	NA
Yes, any kind	3.51 (2.98, 4.14)	3.34 (2.77, 4.03)	NA
Maternal BMI			
Underweight (<18.5 kg/m^2^)	0.86 (0.74, 1.01)	0.90 (0.77, 1.05)	NA
Normal weight (18.5–24.9 kg/m^2^)	Reference	Reference	NA
Overweight (25.0–29.9 kg/m^2^)	1.09 (1.02, 1.16)	1.07 (1.00, 1.14)	NA
Obese (≥30 kg/m^2^)	1.26 (1.15, 1.38)	1.22 (1.11, 1.34)	NA
Maternal diabetes and BMI			
No maternal diabetes and BMI <25 kg/m^2^	Reference	NA	Reference
No maternal diabetes and BMI ≥25 kg/m^2^	1.12 (1.06, 1.19)	NA	1.12 (1.06, 1.18)
Maternal diabetes and BMI <25 kg/m^2^	3.79 (2.91, 4.94)	NA	3.64 (2.79, 4.74)
Maternal diabetes and BMI ≥25 kg/m^2^	3.60 (2.79, 4.65)	NA	3.52 (2.71, 4.55)
Maternal smoking during pregnancy			
No	Reference	Reference	Reference
Yes	0.84 (0.79, 0.90)	0.86 (0.80, 0.92)	0.86 (0.80, 0.92)
Maternal age			
13–29 years	Reference	Reference	Reference
>29 years	1.05 (1.00, 1.10)	1.04 (0.98, 1.10)	1.04 (0.99, 1.10)

^a^Adjusted OR includes size for gestational age, gestational age, maternal diabetes, maternal BMI, maternal smoking habits and maternal age

^b^Adjusted OR includes size for gestational age, gestational age, the combination variable of maternal diabetes and BMI, maternal smoking habits and maternal age

NA, not applicable

A child whose mother had been smoking during pregnancy was less likely to develop type 1 diabetes (crude OR 0.84 [95% CI 0.79, 0.90]; adjusted OR 0.86 [95% CI 0.80, 0.92]), whereas a child whose mother had been either overweight or obese during pregnancy had an increased risk for type 1 diabetes (Table 3). Being born moderately preterm seemed to increase the risk of type 1 diabetes (crude OR 1.11 [95% CI 1.01, 1.23]) and being born very preterm seemed to decrease the risk (crude OR 0.69 [95% CI 0.50, 0.95]), though these significances disappeared in the adjusted model, as did the seemingly increased risk with maternal age. Maternal diabetes was the strongest risk factor for the offspring to develop type 1 diabetes (crude OR 3.51 [95% CI 2.98, 4.14]; adjusted OR 3.34 [95% CI 2.77, 4.03]).

To further investigate whether LGA was an independent risk factor for type 1 diabetes in the offspring, regardless of maternal BMI and maternal diabetes, a new variable was created with four categories depending on whether maternal BMI was < or ≥25 kg/m^2^ and if the mother had diabetes or not. These results showed, as above, that a child born LGA had an increased risk for type 1 diabetes even if their mother had no diabetes and a BMI <25 kg/m^2^ (Table 3).

Information about gestational weight gain was only available for approximately 30% of the mothers (due to missing data on weight at delivery). Therefore, in order to evaluate the possible effect that gestational weight gain may have on the risk for offspring developing type 1 diabetes, models where this variable was included as a confounder were evaluated. These models yielded unchanged ORs for the effect of being SGA on the risk for developing type 1 diabetes. Similarly, for LGA the OR only slightly decreased (<10%) (data not shown). Thus, gestational weight gain was not included as a confounder in the presented analyses.

There were no significant interactions between BMI, parity, maternal smoking, maternal diabetes, pregnancy length and size for gestational age. There was, however, a significant interaction between size for gestational age and maternal age, with older women more often having children who were born LGA.

## Discussion

In this large nationwide case–control study we found that, regardless of sex, being born LGA significantly increased the risk for type 1 diabetes later in life, but being born SGA significantly decreased the risk. These results were the same when looking only at children born at term and regardless of maternal diabetes and BMI. Nevertheless, if the mother was overweight or obese in early pregnancy, this increased the risk of type 1 diabetes, and maternal diabetes was the strongest risk factor.

Our results are in accordance with a large cohort study by Khashan et al [13], which showed that children with type 1 diabetes were more likely to have been born LGA and less likely to have been born SGA, with RR approximately the same as our adjusted OR. However, in their sibling design study, the association between LGA and type 1 diabetes was no longer statistically significant. Another large cohort study from the UK by Goldacre [25] also found an increased risk for type 1 diabetes for children with a high birthweight for gestational age, and a decreased risk for children with a low birthweight. Although it used birthweight z scores instead of size for gestational age, the results of a recent large case–control study from Sweden by Waernbaum et al [26] are in line with our results, showing that a birthweight z score of less than −1 was associated with a decreased risk, and above 1 with an increased risk of type 1 diabetes. However, that study did not include children of mothers with diabetes.

As mentioned above, many studies have used absolute birthweight instead of size for gestational age, although the results may then be harder to interpret since appropriate birthweight is influenced by the actual gestational week in which a baby is born. Nevertheless, our results for absolute birthweight are in line with those for size for gestational age, and with results from two meta-analyses that found an increased risk for type 1 diabetes for children with a birthweight >4000 g [27] or >3500 g [8]. However, these meta-analyses did not find a decreased risk for children with a low birthweight (<2500 g) [8, 27]. The fact that several other studies have failed to show a correlation between birthweight and type 1 diabetes might be due to their small size.

The finding that being born with a low birthweight affects the age at onset of type 1 diabetes is interesting and has not, to the best of our knowledge, been reported previously. However, since there was no significant result for SGA, this raises the question of whether the gestational age plays a role. Also, we have previously reported that maternal BMI affects the age at onset of type 1 diabetes in the offspring, and in the oldest age group nearly 50% of mothers had been underweight, which might explain at least part of the increased proportion of children with a low birthweight.

There seemed to be an increased risk in being born moderately preterm, while being born very preterm was protective; however, the significance disappeared in the adjusted model. Khashan et al [13] reported similar findings in their cohort study, though they used slightly different birth categories, and their results remained in the adjusted models. Waernbaum et al [26] also showed an increased risk for children born moderately premature (32–36 weeks of gestation), whereas being born very prematurely (<32 weeks) was protective against the later development of type 1 diabetes.

It is still unknown how the intrauterine environment might affect the risk of type 1 diabetes. One possible explanation is the accelerator hypothesis which states that overnutrition, weight gain, obesity or insulin resistance give a chronically increased beta cell secretory demand leading to activation of intrinsic beta cell stress pathways that trigger or accelerate autoimmunity [28]. Our finding that LGA increases the risk of type 1 diabetes is in agreement with this but our finding that SGA decreases the risk is not. Children born LGA have most likely been subjected to higher circulating glucose levels, which is the most important energy substrate for a fetus and which makes their pancreatic beta cells secrete more insulin [29]. Insulin is an important fetal growth hormone [10] and studies have shown that active beta cells are more susceptible to destruction [8, 27]. A study has shown that LGA children have increased insulin resistance and beta cell activity at birth [30]. Also, metabolic imprinting during fetal life can be transmitted across generations [31]. Children born LGA have an increased risk for obesity in childhood and early adulthood [32] and childhood adiposity has been linked to an increased risk of type 1 diabetes [33].

To be born SGA has previously been found to increase the risk of hypertension, obesity and type 2 diabetes in adulthood [34]. The fact that there seems to be an inverse relationship with type 1 diabetes is intriguing, especially since children born SGA often show catch-up growth in early life [34] and rapid linear growth in childhood has been shown to increase the risk of type 1 diabetes [11]. Children born SGA, like those born LGA, have been shown to have an increased insulin resistance in childhood, but studies have also shown increased insulin sensitivity at birth in SGA children [30]. In a study with NOD mice, one of the most commonly used animal models of autoimmune diabetes, in utero undernutrition protected the diabetes-prone NOD mice from autoimmune diabetes by reduction of insulitis and beta cell apoptosis [35]. Immune modulation is a mechanism that might help explain the association since low birthweight neonates have defects in cell-mediated and humoral immunity, and these deficits have been documented both in childhood and adolescence [35]. Yet it cannot be ruled out that SGA is merely a substitute for some other underlying protective factor.

In the present study, we did not adjust for gestational weight gain since we only had information on about 30% of the mothers. Also, though gestational weight gain has been suggested as a more important risk factor for LGA children, our results did not show that it increased the risk of type 1 diabetes and it may therefore not be regarded as a confounder or mediator.

Our finding that maternal smoking seemed to protect against type 1 diabetes is interesting and has been previously reported [36]. The mechanism is still largely unknown and requires further investigation, and there is of course always the possibility that maternal smoking is merely a confounding factor. The fact that the seemingly increased risk with maternal age disappeared in the adjusted model might be due to the fact that older mothers often had a higher parity, and with parity the birthweight of the child increases [20]. This is also probably the cause of the significant interaction between size for gestational age and maternal age.

The major strength of our study is that we used a large amount of prospectively collected data (therefore free from recall bias) stored in national registries, allowing us to adjust for many potential confounders. There are, of course, other confounders such as the socioeconomic status of the parents or paternal diabetes or BMI, on which we unfortunately have no information, however, the birthweight of the offspring has been shown to be more associated with the mother’s adiposity [10]. The present study had some limitations. With registry data there is always a risk of misclassification caused by incorrect registration; however, if this is present it is random and not systematic and is the same among cases and controls. As always, when looking at several risk factors there is a risk of multiple comparison error, and when using a large body of material like that used in the present study, there is also a risk of finding clinically irrelevant but statistically significant results. Also, we have missing data on 30% of mothers for maternal BMI which means that we have a smaller population in our multivariate analysis than in the univariate.

In conclusion, size for gestational age of Swedish children affects the risk of type 1 diabetes, with an increased risk if the child is born LGA and a decreased risk if the child is born SGA. To be born LGA is an independent risk factor for type 1 diabetes, irrespective of maternal BMI and diabetes. Thus, reducing the risk for a child being born LGA might to some extent reduce the risk for type 1 diabetes.

## Data Availability

Data are available from the corresponding author on request.

## Abbreviations

AGA: Appropriate for gestational age
LGA: Large for gestational age
MBR: Medical birth register
SGA: Small for gestational age
